# BAM15 Enhances Regorafenib Cytotoxicity in a Canine Osteosarcoma Cell Model

**DOI:** 10.3390/ijms27209121

**Published:** 2026-10-14

**Authors:** Agnieszka Dolnicka, Klaudia Marcinkowska, Bożena Obmińska-Mrukowicz, Vibeke Fosse, Anna Raciborska, Agnieszka Śmieszek

**Affiliations:** 1 Laboratory of Preclinical Research “In VetBio”, Department of Pharmacology and Toxicology, Faculty of Veterinary Medicine, Wroclaw University of Environmental and Life Sciences, Norwida 31, 50-375 Wroclaw, Poland; 2 Department of Clinical Science, Centre for Cancer Biomarkers, University of Bergen, Jonas Lies vei 87, 5021 Bergen, Norway; vibeke.fosse@uib.no; 3 Department of Oncology and Surgical Oncology for Children and Youth, Institute of Mother and Child, Kasprzaka 17a, 01-211 Warsaw, Poland

**Keywords:** canine osteosarcoma, regorafenib, BAM15, mitochondrial uncoupling, metabolic plasticity, comparative oncology

## Abstract

Osteosarcoma is an aggressive primary bone malignancy affecting both humans and dogs, and canine osteosarcoma represents a valuable comparative model for investigating novel therapeutic strategies. Targeting tumour metabolism may enhance the efficacy of existing anticancer treatments. This study investigated whether the mitochondrial uncoupler BAM15 modifies the response of canine D17 osteosarcoma cells to regorafenib. Cells were treated with BAM15 (20 µM) or regorafenib (REG; IC_25_, 8 µM; IC_50_, 16 µM) alone or pretreated with BAM15 before regorafenib exposure. Treatment effects were assessed using viability assays, Annexin V/propidium iodide staining, cell-cycle analysis, Seahorse XF bioenergetic profiling, quantitative PCR, and Western blotting. BAM15 alone did not significantly affect cell metabolic activity. Combined treatment with BAM15 and regorafenib produced a significantly greater reduction in cell metabolic activity than regorafenib alone at IC_50_, whereas no significant difference between treatments was observed at IC_25_. Combined treatment at IC_50_ increased late apoptotic and necrotic populations and disrupted cell-cycle distribution. BAM15 reduced oxidative phosphorylation-derived ATP production without detectable glycolytic compensation but did not further suppress mitochondrial ATP production beyond regorafenib effect alone. Combined treatment also induced transcriptional changes in stress-adaptation-related genes, while protein analysis revealed reduced BAX and BAX/BCL-2 ratio. These exploratory findings provide preliminary evidence that mitochondrial uncoupling by BAM15 may modify the response to regorafenib and support further mechanistic investigation and validation in additional tumour and non-malignant canine models.

## 1. Introduction

Osteosarcoma occurs in dogs as a spontaneous malignancy and shows striking similarities in canine and human pathophysiology, biological behaviour, diagnosis, prognosis and treatment options [1]. Therefore, dogs serve as highly relevant comparative models in preclinical studies, accelerating the development of essential, life-saving therapeutics, enabling earlier assessment of drug safety and efficacy in a naturally occurring disease context [2]. Translational osteosarcoma research provides valuable insights into tumour biology, mechanisms of drug resistance, and treatment responses that are relevant to human clinical practice. This comparative, bidirectional approach advances veterinary care and has the potential to improve human cancer treatment strategies significantly [1,3].

Regorafenib (REG), an established anti-cancer drug in human medicine, represents a compelling candidate for repurposing in veterinary oncology. The drug is a multikinase inhibitor with anti-angiogenic and anti-proliferative effects [4]. It has emerged as a candidate therapy in human osteosarcoma, demonstrating limited but promising activity in clinical studies [5]. A randomised, double-blind, placebo-controlled study by Davis et al. demonstrated that regorafenib prolonged disease-free survival in adult patients with metastatic osteosarcoma compared with placebo [6]. Clinically, the drug has been approved in humans for the treatment of metastatic colorectal cancer, gastrointestinal stromal tumours (GIST), and hepatocellular carcinoma after progression on standard therapies [7]. In canines, the drug has been tested for its ability to reduce conjunctival scarring in postoperative wounds following glaucoma filtration surgery. Still, it has not yet been tested in veterinary oncology [8].

The limitations of current osteosarcoma therapies, together with the frequent emergence of drug resistance, underscore the need for alternative strategies that target broader and more fundamental vulnerabilities of tumour cells. Among these, altered energy metabolism and mitochondrial dysfunction have emerged as key determinants of osteosarcoma progression, survival, and therapeutic resistance. Targeting tumour bioenergetics represents a promising complementary approach to conventional signalling-based therapies [9].

In this context, we investigated BAM15, a mitochondrial uncoupler, as a candidate agent capable of modulating mitochondrial function and disrupting metabolic homeostasis, with the potential to enhance anti-tumour activity in osteosarcoma models. BAM15, also known as N5, N6-Bis(2-fluorophenyl)[2,3]oxadiazolo [4,5-b]pyrazine-5,6-diamine, modulates cell metabolism by disrupting mitochondrial membrane potential [10,11]. As a protonophore, BAM15 reduces mitochondrial coupling efficiency, thereby compromising ATP production [12,13]. Due to rapid proliferation, cancer cells have higher energy requirements and depend more on mitochondrial metabolism [14]. This mechanism suggests that BAM15 may act more selectively on cancer cells, potentially improving the effects of anti-cancer agents. Moreover, BAM15 is a novel agent under investigation for its potential in obesity, diabetes, sepsis, cardiovascular disease, and cancer treatment [10,13,15].

In this exploratory in vitro study, we investigated the dose-dependent effects of regorafenib in D17 canine osteosarcoma cells and assessed whether BAM15 modifies their response to treatment. This approach was informed by evidence that cellular metabolic context may influence responses to targeted therapies [16]. Although limited to a single cell model, the study provides a starting point for further mechanistic investigation of mitochondrial uncoupling in osteosarcoma, with comparative oncology offering a broader context for future research.

## 2. Results

### 2.1. Regorafenib Reduces the Viability of D17 Canine Osteosarcoma Cells in a Concentration-Dependent Manner, with BAM15 Enhancing Metabolic Decline at IC_50_

**Figure 1:**
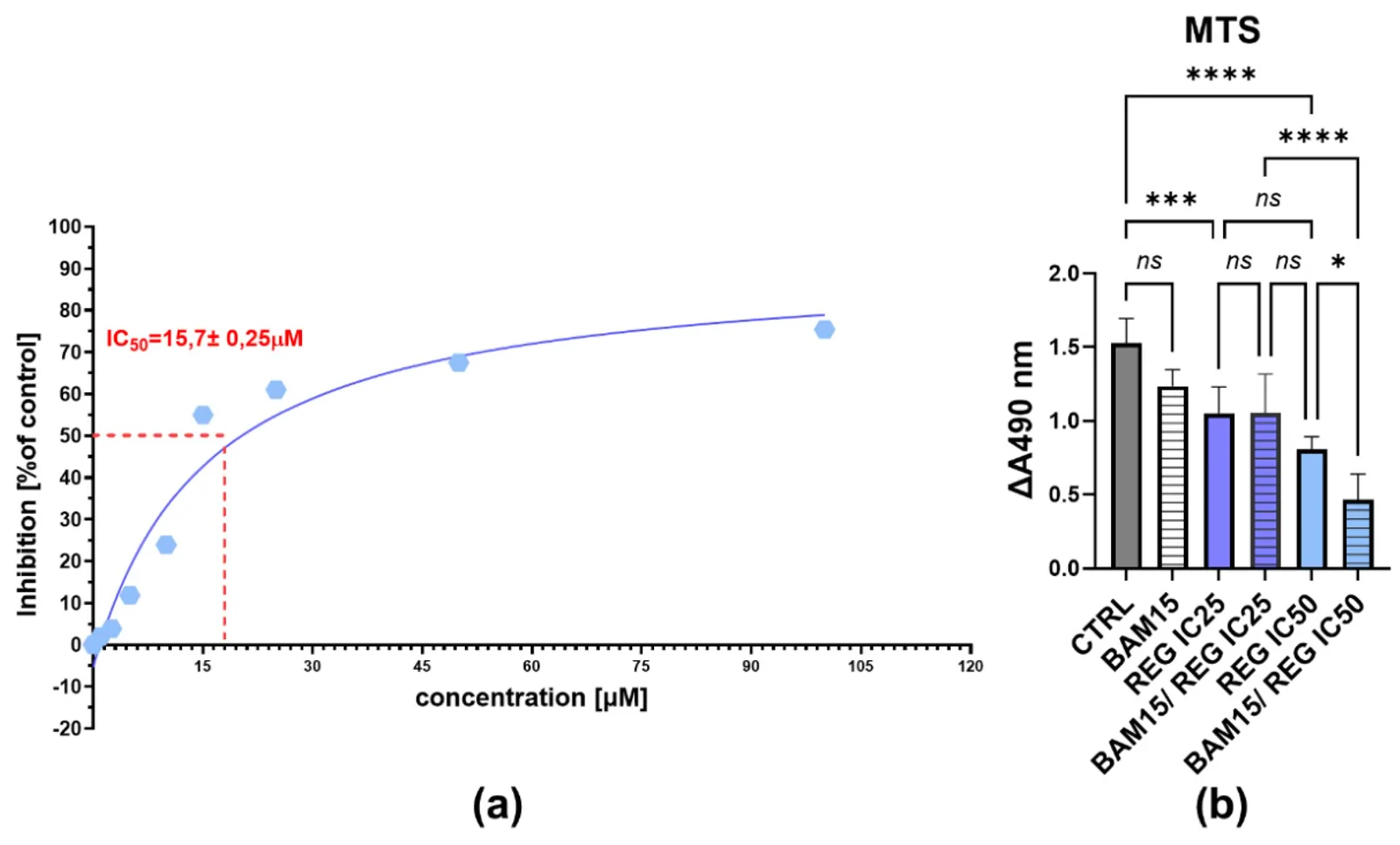
Cytotoxic effects of regorafenib alone and in combination with BAM15 in canine D17 osteosarcoma cells. Dose–response curve of D17 canine osteosarcoma cell viability following 48 h exposure to increasing concentrations of regorafenib (**a**) and MTS assay results (**b**) showing the effect of BAM15, regorafenib (REG; IC_25_ and IC_50_), and their combination on the metabolism of the cells after 48 h of treatment. Data are presented as mean ± SD from three independent biological experiments (*n* = 3). Statistical significance was assessed using ANOVA followed by a post hoc test. * *p* < 0.05, *** *p* < 0.001, **** *p* < 0.0001; *ns*—not significant.

The IC50 value obtained for regorafenib in D17 cells was 15.7 ± 0.25 μM, as determined from the dose–response curve, reflecting concentration-dependent inhibition of cell viability (Figure 1a).

Evaluation of regorafenib and BAM15, tested alone and in combination, showed that regorafenib reduced the viability of D17 cells in a concentration-dependent manner, with a more pronounced effect observed at IC_50_ compared to IC_25_. Compared with the control, BAM15 treatment alone showed no cytotoxicity, and BAM15 pretreatment did not modify the cytotoxic response to regorafenib at IC_25_. Notably, pretreatment with BAM15 followed by regorafenib at its IC_50_ significantly decreased cell viability compared with regorafenib alone at its IC_50_ (Figure 1b).

### 2.2. Regorafenib at IC25 Does Not Alter the Apoptotic Profile, Whereas IC50 Induces Marked Cytotoxicity That Is Enhanced by BAM15 in D17 Canine Osteosarcoma Cells

**Figure 2:**
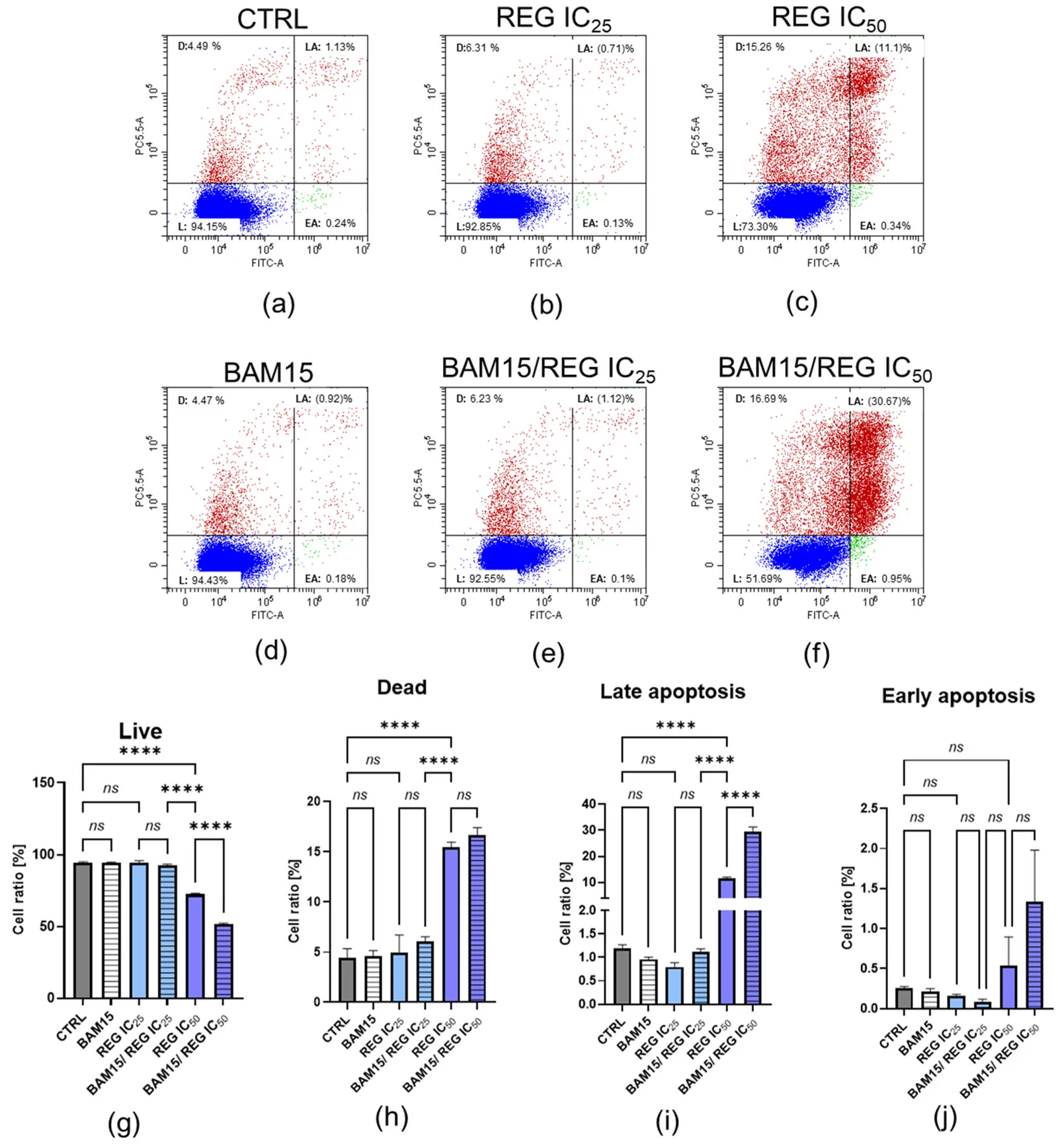
Annexin V/PI analysis of apoptosis in osteosarcoma canine D17 cells treated with regorafenib and BAM15. Representative dot plots of Annexin V–FITC/propidium iodide (PI) staining in canine osteosarcoma D17 cells following treatment with regorafenib (IC25 and IC50), BAM15 alone, or BAM15 pre-treatment followed by regorafenib (IC_25_ and IC_50_). Quadrant analysis distinguishes viable (Annexin V^−^/PI^−^), early apoptotic (Annexin V^+^/PI^−^), late apoptotic (Annexin V^+^/PI^+^), and necrotic (Annexin V^−^/PI^+^) cell populations. Representative dot plots are shown for (**a**) vehicle control, (**b**) REG IC25, (**c**) REG IC50, (**d**) BAM15 alone, (**e**) BAM15 pretreatment followed by REG IC25, and (**f**) BAM15 pretreatment followed by REG IC50. Panels (**g**–**j**) show the percentages of (**g**) viable, (**h**) necrotic, (**i**) late apoptotic, and (**j**) early apoptotic cells, respectively. Quantitative data are shown below the representative plots and presented as mean ± SD from three independent biological experiments (*n* = 3). Statistical significance was assessed using ANOVA followed by a post hoc test; **** *p* < 0.0001; *ns*—not significant.

The analysis of the effects of regorafenib, BAM15, and their combination on cell survival and apoptosis in canine osteosarcoma D17 cells revealed that BAM15 alone did not significantly affect cell metabolism or the distribution of apoptotic populations compared with the control (Figure 2a,d,g–j). Similarly, treatment with regorafenib at the IC25 concentration did not induce significant changes in the apoptotic profile of D17 cells, and this pattern remained unchanged following BAM15 pre-treatment (Figure 2a,b,e,g–j).

In contrast, regorafenib at the IC_50_ concentration exerted a marked cytotoxic effect, manifested by a significant increase in the proportion of late apoptotic cells, along with an elevation in the early apoptotic fraction (Figure 2c). Notably, pre-treatment with BAM15 further enhanced this effect, resulting in a statistically significant increase in late apoptosis in D17 cells (Figure 2f,i). An increased proportion of necrotic cells additionally reflected the cytotoxic response to regorafenib at IC_50_ (Figure 2c,h). The accumulation of cells in late apoptosis, together with an increased necrotic fraction, reflects the strong cytotoxic effect of regorafenib at IC_50_. Moreover, BAM15 increased the sensitivity of D17 cells to the drug, resulting in rapid progression toward irreversible cell death (Figure 2f,h).

Collectively, these results indicate that while BAM15 alone does not affect the survival or apoptotic status of D17 cells, it amplifies regorafenib-induced cytotoxicity at higher drug concentrations (Figure 2).

### 2.3. Regorafenib at IC25 Induces G0/G1 Cell-Cycle Arrest, Whereas IC50 Promotes Cell-Cycle Disruption That Is Enhanced by BAM15 in D17 Canine Osteosarcoma Cells

**Figure 3:**
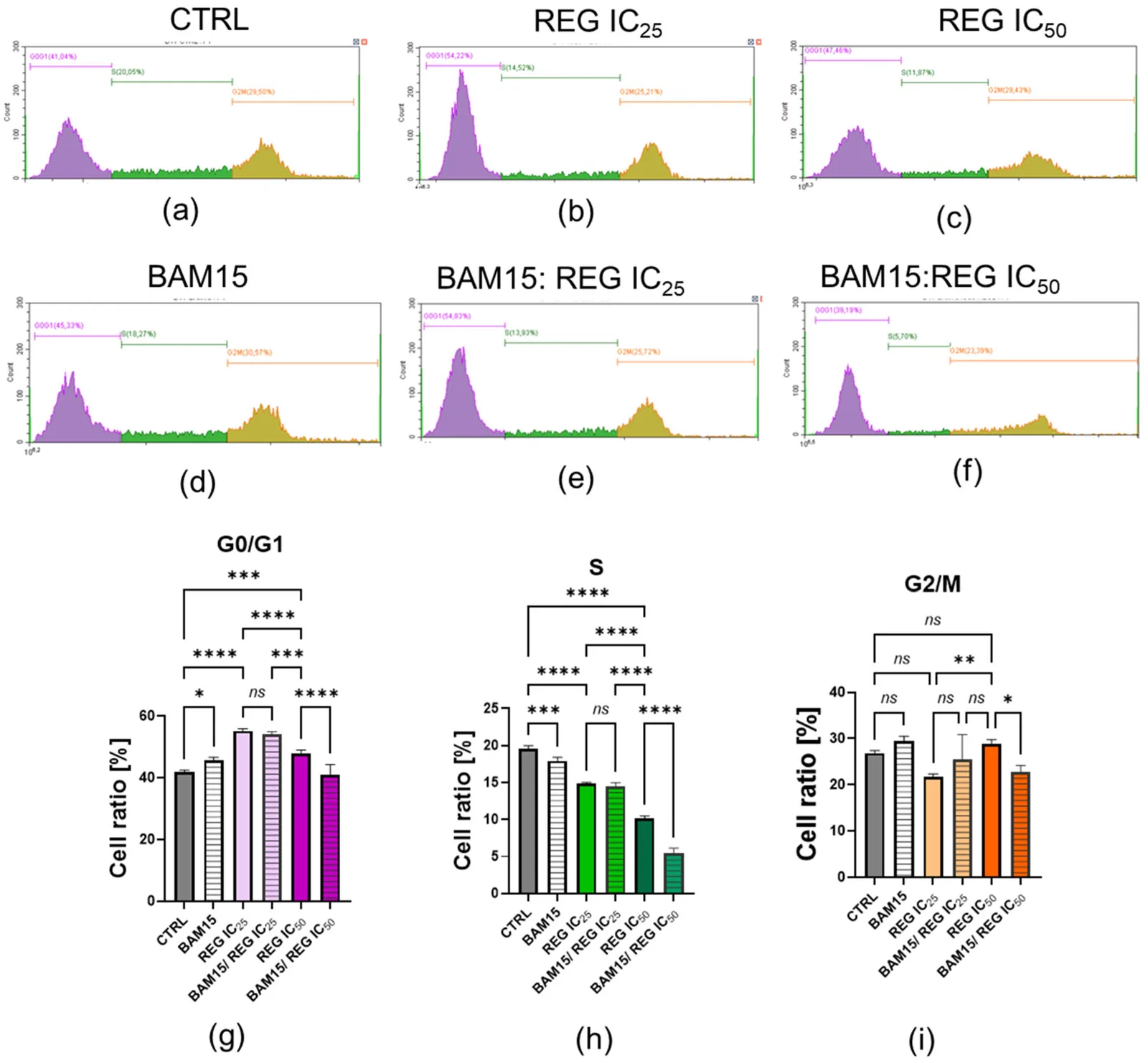
Effect of BAM15, regorafenib and their combination on cell-cycle distribution of canine osteosarcoma D17 cells. Representative flow cytometry histograms showing Propidium Iodide staining of D17 cells treated with vehicle control (CTRL) (**a**), regorafenib at IC_25_ (**b**), regorafenib at IC50 (**c**), BAM15 (20 µM) (**d**), BAM15 combined with regorafenib IC_25_ (**e**), and BAM15 combined with regorafenib IC_50_ (**f**). Quantitative data showing the percentages of cells in the G0/G1 (**g**), S (**h**), and G2/M (**i**) phases are presented as mean ± SD from three independent biological experiments (*n* = 3). Statistical significance was assessed relative to control or between indicated groups (* *p* < 0.05, ** *p* < 0.01, *** *p* < 0.001, **** *p* < 0.0001; *ns*, not significant).

BAM15 treatment resulted in a significant increase in the proportion of D17 canine osteosarcoma cells in the G0/G1 phase, accompanied by a marked reduction in the proliferating S-phase population. In contrast to the control, BAM15 did not affect the cells’ shift into the G2/M fraction (Figure 3g–i).

Regorafenib treatment at both IC_25_ and IC_50_ concentrations induced a significant accumulation of cells in the G0/G1 phase. Notably, a more pronounced G0/G1 arrest was observed at the lower concentration of the drug (Figure 3g). The regorafenib-induced G0/G1 arrest was associated with a decrease in the proportion of S-phase cells (Figure 3h). At the same time, regorafenib treatment at IC_50_ additionally increased the fraction of cells in the G2/M phase compared to IC_25_.

Analysis of the combined treatment confirmed that BAM15 potentiated the toxic effect of regorafenib only at the IC_50_, as evidenced by a further reduction in the S-phase population and a decreased proportion of cells in the G2/M phase (Figure 3i).

Overall, the data suggest that regorafenib at IC_25_ causes G0/G1 arrest of canine D17 cells, whereas treatment of the cells with the drug at IC_50_ results in broader alterations in cell-cycle distribution, which are also enhanced by BAM15 action (Figure 3g–i).

### 2.4. Glycolysis-Supported ATP Production Is Preserved Despite OXPHOS Impairment Induced by BAM15 and Regorafenib in D17 Canine Osteosarcoma Cells

**Figure 4:**
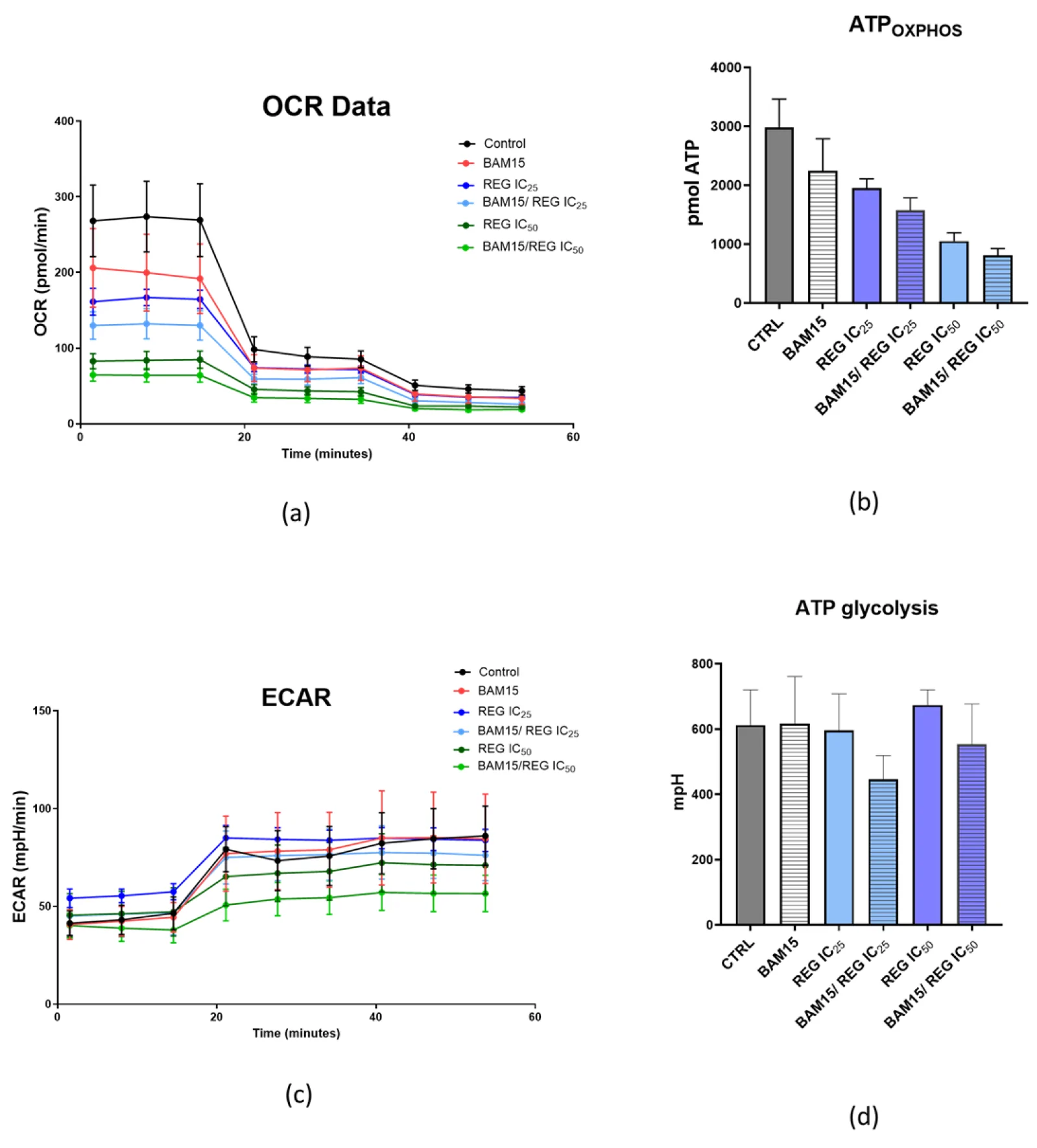
Effects of BAM15 and regorafenib on mitochondrial respiration and glycolytic activity in canine osteosarcoma D17 cells. (**a**) Oxygen consumption rate (OCR) measured over time using the Seahorse XF analyser (Agilent Technologies, Santa Clara, CA, USA) following treatment with BAM15 (20 µM), regorafenib at IC_25_ or IC_50_, or their combinations, compared to control cells. (**b**) Quantification of ATP production derived from oxidative phosphorylation (ATP_OXPHOS_). (**c**) Extracellular acidification rate (ECAR) measured as an indicator of glycolytic activity. (**d**) Quantification of ATP production derived from glycolysis. Data are presented as mean ± SD from two independent biological experiments (*n* = 2), each performed in technical duplicate. No inferential statistics or significance annotations are reported. Regorafenib induces dose-dependent cytotoxicity- and adaptation-related transcriptional responses in D17 canine osteosarcoma cells, differentially altered by BAM15.

Real-time analysis of ATP production in canine osteosarcoma D17 cells (Figure 4) revealed that exposure to BAM15, as well as regorafenib at IC_25_ and IC_50_ concentrations applied individually, resulted in reduction in ATP synthesis from oxidative phosphorylation (OXPHOS; Figure 4a,b). In contrast, glycolysis-supported ATP production remained unaffected under these conditions. Furthermore, BAM15 pretreatment followed by regorafenib at both IC_25_ and IC_50_ led to a modest decrease in ATP synthesis from OXPHOS and glycolysis (Figure 4b,d). These observations are preliminary and require confirmation in additional independent experiments. The metabolic profile observed after BAM15 treatment is consistent with its established role as a mitochondrial uncoupler, which reduces OXPHOS efficiency without causing a profound depletion of cellular ATP pools or a compensatory upregulation of glycolytic ATP production. The metabolic profile observed under experimental conditions indicates a selective vulnerability of OXPHOS-dependent ATP synthesis in D17 cells. At the same time, glycolysis-supported energy production in D17 remains largely resilient to both BAM15 and regorafenib exposure, which is consistent with the expected bioenergetic response to mitochondrial uncoupling and targeted kinase inhibition (Figure 4a–d).

**Figure 5:**
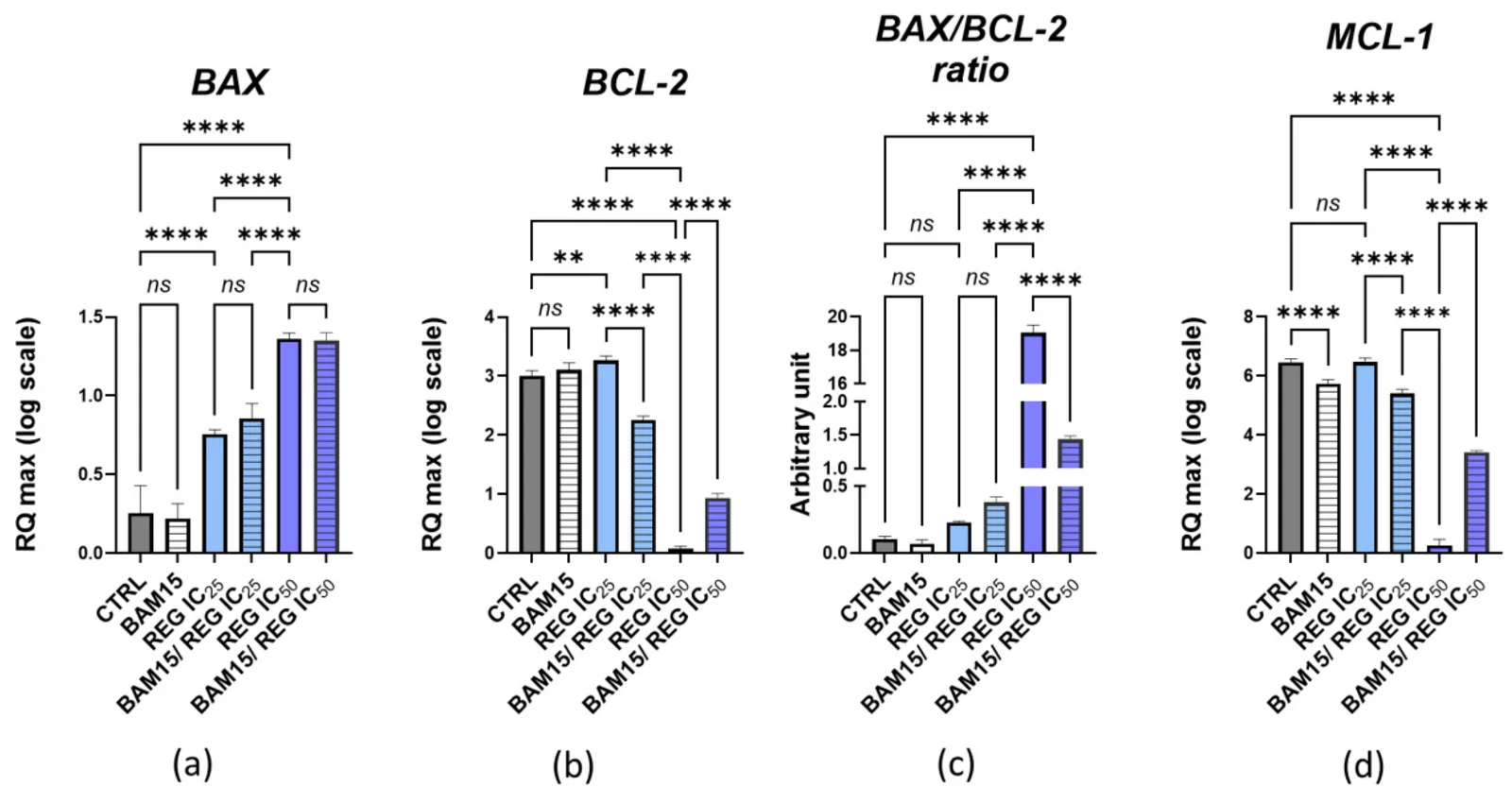
Effect of BAM15 and regorafenib on the expression of apoptosis-related genes in canine osteosarcoma D17 cells. Relative mRNA expression levels of the pro-apoptotic gene BAX (**a**), anti-apoptotic genes BCL-2 (**b**) and MCL-1 (**d**), and the BAX/BCL-2 ratio (**c**) were determined following treatment with BAM15 (20 µM), regorafenib at IC_25_ or IC_50_, and their combinations. Gene expression was normalised to a reference gene and is presented as log-transformed RQmax values. Data are shown as mean ± SD from three independent biological experiments (*n* = 3), each performed in technical triplicate. Statistical significance between groups is indicated ( ** *p* < 0.01, **** *p* < 0.0001; *ns*, not significant).

Analysis of apoptosis-related transcripts in the D17 cell line demonstrated that regorafenib markedly altered the balance between pro- and anti-apoptotic signalling in a dose-dependent manner, while BAM15 modulated these effects (Figure 5).

BAX expression was significantly upregulated following regorafenib treatment, with increased mRNA levels observed at both IC25 and IC50 concentrations. BAM15 alone did not significantly affect BAX expression in D17 cells, and co-treatment with BAM15 and regorafenib did not further enhance BAX levels compared with regorafenib alone, indicating that BAX induction was primarily driven by regorafenib (Figure 5a).

At the IC25 concentration, regorafenib treatment was associated with increased accumulation of the anti-apoptotic BCL-2 transcript in D17 cells, while pre-treatment with BAM15 reduced BCL-2 mRNA expression. In contrast, BCL-2 expression in D17 cells was significantly decreased by regorafenib at the IC50 concentration, whereas pre-treatment with BAM15 partially attenuated this effect (Figure 5b).

Analysis of the BAX/BCL-2 ratio revealed a modest increase in the pro-apoptotic balance at the IC25 concentration of regorafenib following BAM15 pre-treatment. In contrast, regorafenib alone at IC50 produced the strongest pro-apoptotic shift, whereas BAM15 pre-treatment was associated with a relative reduction in this effect (Figure 5c).

Changes in the BAX/BCL-2 ratio were paralleled by alterations in MCL-1 mRNA (Figure 5d). D17 treated only with BAM15 treatment had significantly reduced MCL-1 transcript levels compared with the control culture, indicating an intrinsic effect of mitochondrial uncoupling on this anti-apoptotic regulator. In contrast, D17 treatment with regorafenib at the IC25 concentration alone did not alter MCL-1 expression in relation to the control; however, BAM15 pre-treatment resulted in a significant decrease in MCL-1 mRNA levels. The strongest suppression of MCL-1 expression was observed with regorafenib alone at IC50, while BAM15 pre-treatment was associated with a partial mitigation of this effect.

**Figure 6:**
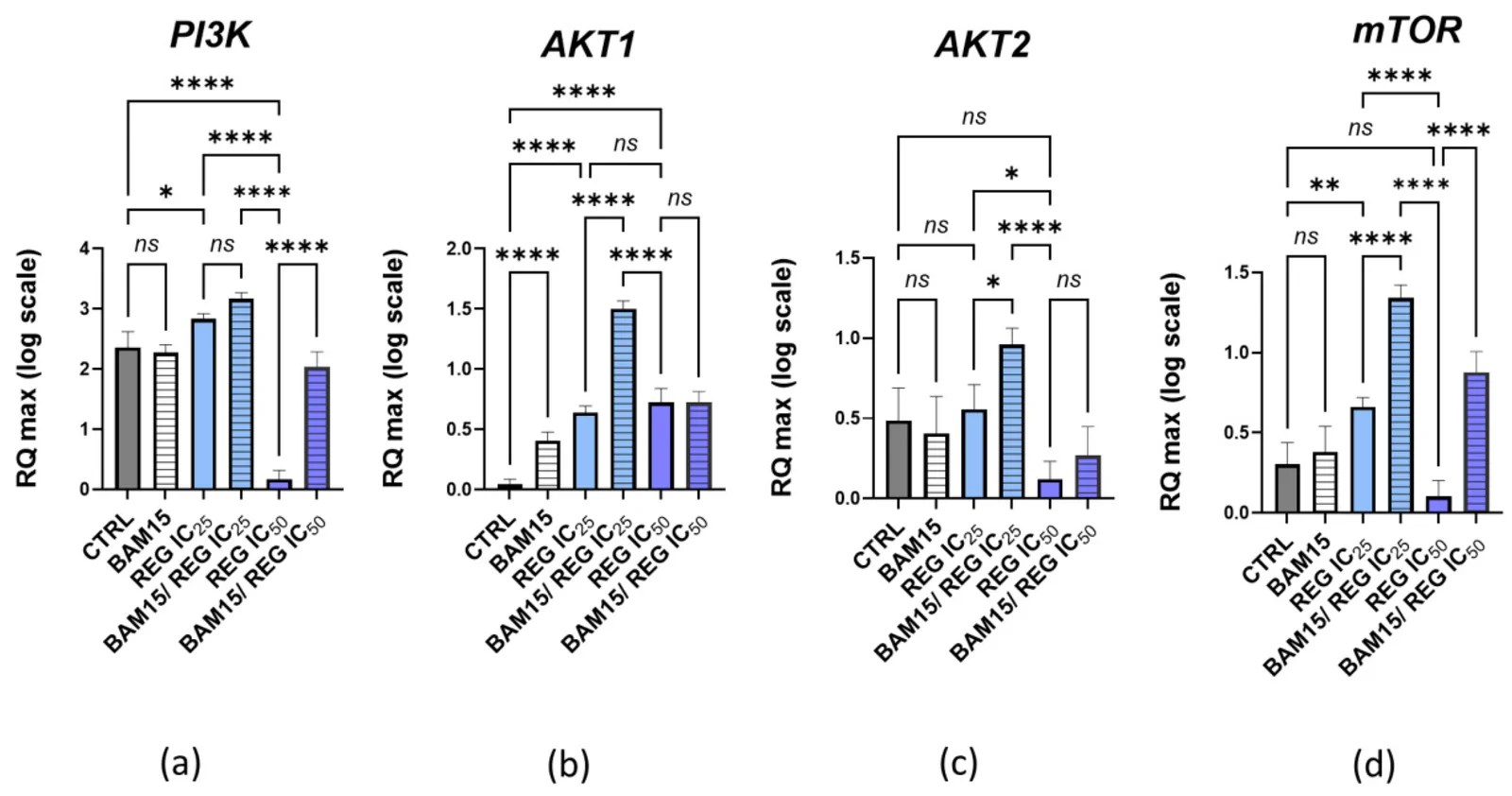
Effect of BAM15, regorafenib, and their combination on the expression of PI3K/AKT/mTOR pathway genes in D17 osteosarcoma cells. Gene expression levels of PI3K (**a**), AKT1 (**b**), AKT2 (**c**), and mTOR (**d**) were analysed by quantitative PCR in D17 cells under control conditions (CTRL), BAM15 alone (20 µM), regorafenib alone (REG; IC_25_ or IC_50_), or a combination of BAM15 with regorafenib (BAM15/REG IC_25_ and BAM15/REG IC_50_). In the combination groups, cells were pretreated with BAM15 for 16 h, followed by regorafenib exposure for 48 h. Gene expression was normalised to a reference gene and is presented as log-transformed RQmax values. Data are shown as mean ± SD from three independent biological experiments (*n* = 3), each performed in technical triplicate. Statistical significance was determined using appropriate statistical tests and is indicated as *p* < 0.05 (*), *p* < 0.01 (**), *p* < 0.0001 (****); *ns*, not significant.

Analysis of the expression of genes related to the PI3K–AKT–mTOR signalling pathway also indicated a dose-dependent effect of regorafenib on the transcriptional activity of its key components and a modulatory effect of BAM15 (Figure 6).

Treatment of D17 cells with regorafenib at the IC_25_ concentration resulted in a significant upregulation of PI3K, AKT1, and mTOR mRNA levels compared with control cultures (Figure 6a,b,d). Moreover, pre-treatment with BAM15 followed by regorafenib exposure at the IC_25_ concentration resulted in a significant upregulation of AKT1 and mTOR transcripts, suggesting their involvement in modulating the adaptive response of D17 cells to sub-cytotoxic stress. This transcriptional response is consistent with the activation of pro-survival mechanisms and correlates with the increased mRNA expression of the anti-apoptotic factor BCL-2. Moreover, BAM15 alone significantly affected only AKT1 expression.

In contrast, exposure to regorafenib at the IC_50_ concentration led to marked suppression of PI3K and mTOR expression, which is consistent with pathway inhibition under cytotoxic conditions (Figure 6a,d). Notably, BAM15 pre-treatment partially restored expression of both genes in cells treated with regorafenib at IC_50_, indicating a modulatory effect of mitochondrial uncoupling on mRNA levels of these markers (Figure 6a,d). Regorafenib-induced upregulation of AKT1 expression in D17 at IC_50_ concentration resulted in comparable transcript levels to those observed, and was not significantly influenced by prior BAM15 exposure (Figure 6b).

AKT2 expression was not significantly altered following treatment with BAM15 alone or regorafenib at either IC_25_ or IC_50_ when compared with control cells (Figure 6c). However, treatment with regorafenib at IC_50_ resulted in a significant decrease in AKT2 expression compared with IC_25_. In contrast, BAM15 pre-treatment combined with regorafenib at IC_25_ led to a significant upregulation of AKT2, suggesting activation of a compensatory pro-survival and metabolic adaptation response. This effect was not observed at the IC_50_ concentration, where BAM15 pre-treatment did not modify AKT2 expression, consistent with the predominance of regorafenib-induced cytotoxicity at higher drug doses (Figure 6c).

**Figure 7:**
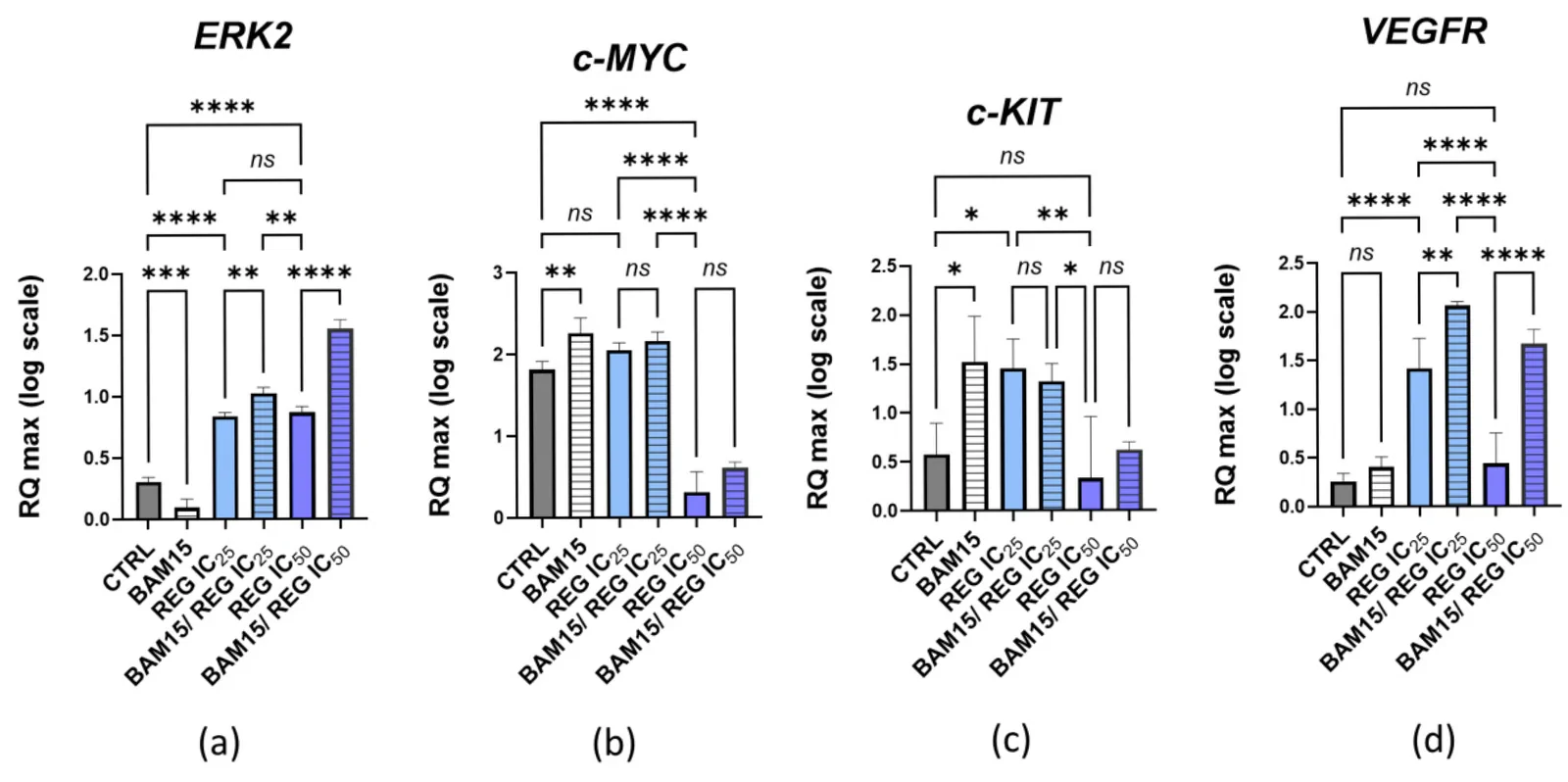
Effect of BAM15, regorafenib, and their combination on the expression of ERK2, c-MYC, c-KIT, and VEGFR in D17 osteosarcoma cells. Relative mRNA expression levels of ERK2 (**a**), c-MYC (**b**), c-KIT (**c**), and VEGFR (**d**) were quantified by qPCR in D17 cells under control conditions (CTRL), BAM15 alone (20 µM), regorafenib alone (REG; IC_25_ or IC_50_), or combined BAM15 and regorafenib treatment (BAM15/REG IC_25_ and BAM15/REG IC_50_). In the combination groups, cells were pretreated with BAM15 for 16 h, followed by regorafenib exposure for 48 h. Expression levels are presented as log-transformed RQmax values. Data are shown as mean ± SD from three independent biological experiments (*n* = 3), each performed in technical triplicate. Statistical significance was assessed using appropriate statistical tests and is indicated as *p* < 0.05 (*), *p* < 0.01 (**), *p* < 0.001 (***), *p* < 0.0001 (****); *ns*—not significant.

Further analysis of the stress-responsive signalling profile revealed that D17 cells exposed to BAM15 alone exhibited a significant reduction in ERK2 expression (Figure 7a). In contrast, regorafenib treatment at both IC_25_ and IC_50_ concentrations led to a significant upregulation of ERK2 expression, but in a dose-independent manner. Notably, BAM15 pre-treatment further enhanced ERK2 expression in regorafenib-treated cells at both concentrations (Figure 7a).

Moreover, compared with control cells, BAM15 alone significantly upregulated c-MYC and c-KIT expression (Figure 7b,c). Regorafenib exposure at IC_25_ similarly significantly increased c-MYC and c-KIT transcript levels, and this effect was not further modified by BAM15 pre-treatment, suggesting that mitochondrial uncoupling does not interfere with the adaptive transcriptional response observed under IC_25_ treatment conditions. In contrast, regorafenib at IC_50_ led to reductions in both c-MYC and c-KIT expression; however, a statistically significant decrease compared with control was observed only for c-MYC. In turn, BAM15 pre-treatment did not alter c-MYC or c-KIT expression at IC50, which is consistent with a cytotoxic treatment profile of the drug at high concentration (Figure 7b,c).

Analysis of VEGFR expression revealed that BAM15 treatment alone did not significantly affect transcript levels compared with control cells (Figure 7d). In contrast, regorafenib exposure at IC_25_ resulted in a significant upregulation of VEGFR. At the same time, BAM15 pre-treatment further enhanced VEGFR expression in cells treated with regorafenib at IC_25_. At IC_50_, regorafenib treatment alone did not significantly alter VEGFR expression compared with control cells. In turn, BAM15 pre-treatment resulted in a significant increase in VEGFR transcript levels, which again points to the engagement of compensatory, stress-associated receptor pathways (Figure 7d).

**Figure 8:**
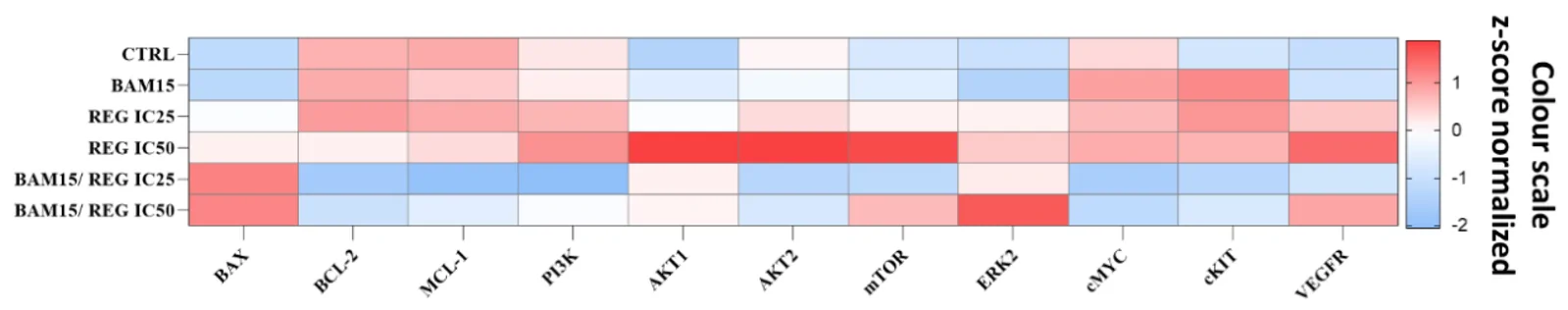
Heatmap representation of gene expression changes in D17 osteosarcoma cells following treatment with BAM15, regorafenib, and their combination. The heatmap illustrates the relative expression patterns of genes associated with apoptosis (BAX, BCL-2, MCL-1), PI3K/AKT/mTOR and MAPK signalling (PI3K, AKT1, AKT2, mTOR, ERK2), oncogenic regulation (c-MYC), and receptor tyrosine kinase signalling (c-KIT, VEGFR) in D17 cells under control conditions (CTRL), BAM15 alone (20 µM), regorafenib alone (REG IC_25_ or REG IC_50_), or combined BAM15 and regorafenib treatment (BAM15/REG IC_25_ and BAM15/REG IC_50_). In the combination groups, cells were pretreated with BAM15 for 16 h, followed by regorafenib exposure for 48 h. Gene expression values were normalised and converted to Z-scores for visualisation. Red indicates higher relative expression, while blue indicates lower relative expression, as shown by the colour scale.

To summarise, the analysis revealed dose-dependent remodelling of gene expression profiles in D17 cells treated with regorafenib, with BAM15 affecting the expression of selected genes and modulating regorafenib’s effects (Figure 8). IC_25_ exposure, with or without BAM15 pre-treatment, was associated with coordinated upregulation of adaptive survival- and receptor-related genes, whereas IC_50_ treatment induced a pro-apoptotic expression pattern marked by c-MYC and c-KIT suppression and increased BAX expression, while BAM15 pre-treatment selectively restored genes associated with adaptation to the stress (Figure 5, Figure 6, Figure 7 and Figure 8).

### 2.5. Regorafenib and BAM15 Differentially Alter BAX and BCL-2 Protein Levels in D17 Canine Osteosarcoma Cells

**Figure 9:**
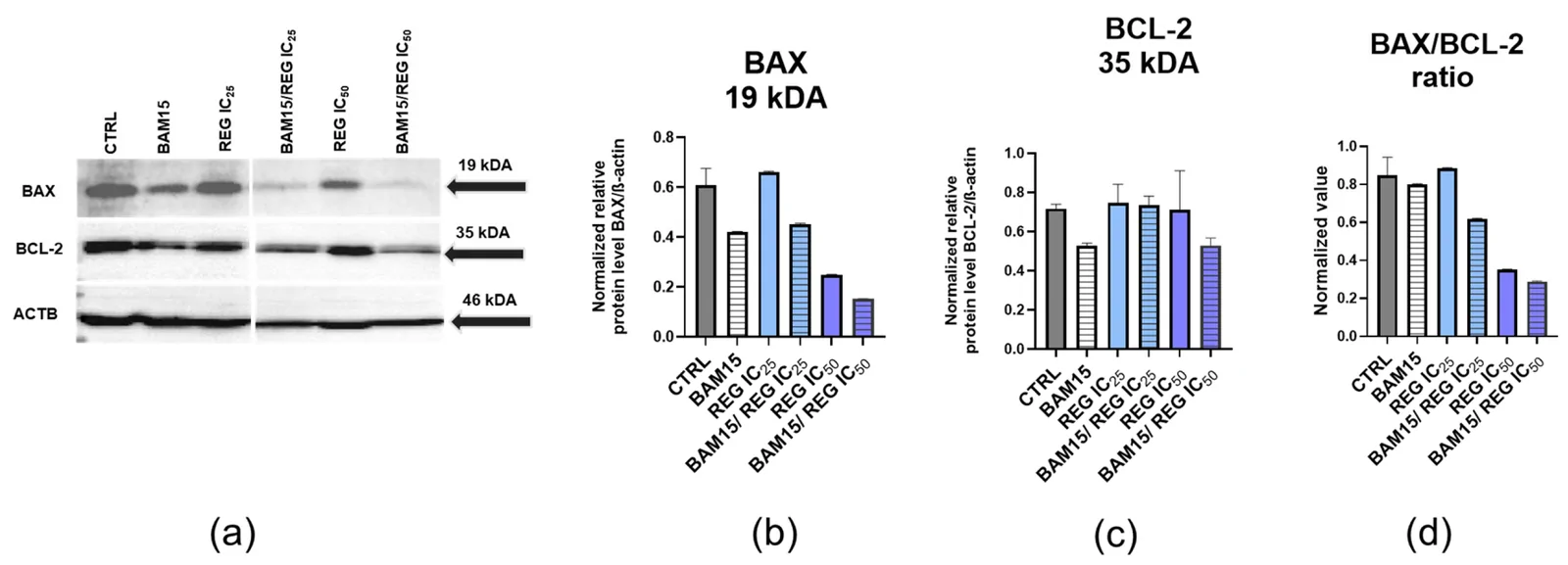
Effect of BAM15, regorafenib, and their combination on BAX and BCL-2 protein levels in D17 osteosarcoma cells. Representative Western blot images of BAX, BCL-2, and β-actin (ACTB) (**a**), and densitometric quantification of BAX (**b**), BCL-2 (**c**), and the BAX/BCL-2 ratio (**d**) in D17 cells under control conditions (CTRL), BAM15 alone (20 µM), regorafenib alone (REG; IC_25_ or IC_50_), or combined BAM15 and regorafenib treatment (BAM15/REG IC_25_ and BAM15/REG IC_50_). In the combination groups, cells were pretreated with BAM15 for 16 h, followed by regorafenib exposure for 48 h. BAX and BCL-2 protein levels were normalised to ACTB, and the BAX/BCL-2 ratio was calculated from the normalised values. Data are presented as mean ± SD from two independent biological experiments (*n* = 2), each performed in technical duplicate. No inferential statistics or significance annotations are reported.

Western blot analysis showed variation in BAX protein levels across treatment groups, while BCL-2 levels appeared relatively similar (Figure 9a–d). BAM15 alone reduced BAX levels compared with the control but did not affect BCL-2 levels or the BAX/BCL-2 ratio. Regorafenib at IC_25_ did not alter BAX or BCL-2 levels relative to the control. However, the combination of BAM15 and regorafenib at IC_25_ reduced both BAX levels and the BAX/BCL-2 ratio compared with regorafenib at IC_25_ alone, while BCL-2 levels remained unchanged. Regorafenib at IC_50_ markedly reduced BAX levels and the BAX/BCL-2 ratio, and adding BAM15 did not significantly enhance these effects. The observed changes were only partially consistent with the Annexin V/PI staining results, which indicated increased late apoptosis and necrosis following treatment with REG IC_50_, particularly in combination with BAM15 (Figure 2). However, these effects were not accompanied by an increased BAX/BCL-2 ratio (Figure 9d). Moreover, the protein-level changes were not fully consistent with the corresponding transcriptional profiles (Figure 5a–c), suggesting that changes in BAX and BCL-2 transcript abundance were not directly translated into alterations in their protein levels. However, observations from Western blot are based on two independent biological experiments and thus should be considered as preliminary.

## 3. Discussion

Canine osteosarcoma represents a valuable comparative model for investigating therapeutic strategies relevant to both veterinary and human oncology [1,3]. Given the biological similarities between canine and human osteosarcoma, studies using canine tumour models can provide insight into mechanisms of treatment response and resistance while also identifying strategies with potential cross-species translational relevance [1]. Among these approaches, targeting altered mitochondrial function and metabolic plasticity may complement signalling-based therapies and enhance their antitumour activity [9,17].

Repurposing agents used in human oncology has become an attractive strategy in veterinary oncology, especially when paired with rational combinations that exploit fundamental tumour vulnerabilities, such as altered mitochondrial function and metabolic plasticity [18]. Previous studies suggest that combining agents with complementary mechanisms of action may improve anti-tumour responses without increasing individual drug doses, providing a rationale for further evaluation of such approaches in canine oncology, including their efficacy and tolerability [18,19,20].

In light of these considerations, we evaluated the anti-tumour activity of regorafenib, a multi-kinase inhibitor approved for human oncology, using the D17 canine osteosarcoma cell model. We investigated whether BAM15, through its mitochondrial uncoupling activity, could modify the response of D17 canine osteosarcoma cells to regorafenib in vitro. The key finding of the present study is that BAM15 pretreatment enhanced the cytotoxic response of D17 cells to regorafenib at the IC_50_ concentration. This effect was reflected by a greater reduction in cell viability and an increased proportion of late apoptotic and necrotic cells, indicating that mitochondrial uncoupling can modify the cellular response to regorafenib under cytotoxic treatment conditions. Importantly, at IC_25_, however, combined treatment with regorafenib and BAM15 did not produce a significantly greater reduction in metabolic activity than regorafenib alone, suggesting that the interaction between mitochondrial uncoupling and regorafenib depends on the intensity of treatment-induced stress.

Regorafenib, a multi-kinase inhibitor widely used in human oncology for the treatment of metastatic colorectal cancer [21], gastrointestinal stromal tumours [22], and hepatocellular carcinoma [23], has demonstrated the ability to inhibit angiogenesis, tumour proliferation, and survival signalling across multiple tumour types. Its broad mechanism of action includes simultaneous inhibition of angiogenic (VEGFR1–3, TIE2), stromal (PDGFR, FGFR), and oncogenic kinases (BRAF, KIT), enabling multi-level interference with tumour growth and vascular support, as supported by both preclinical and clinical studies [24].

In veterinary medicine, regorafenib was used as eye drops to reduce intraocular pressure and improve postoperative bleb formation after glaucoma filtration surgery in dogs. It also reduced postoperative scarring and vascularisation, as shown by lower subconjunctival collagen and vessel densities and fewer vimentin-, TGF-β-, PCNA-, and α-SMA-positive cells compared with the vehicle-treated control [8]. However, the anti-tumour activity of regorafenib has not been formally assessed in canine cancers, including osteosarcoma, where novel targeted strategies remain urgently needed. Systemic regorafenib pharmacokinetics have not been clinically established in canine oncology. Nevertheless, non-clinical toxicokinetic studies in Beagle dogs demonstrated measurable systemic exposure following repeated oral dosing, with C_max_ values of approximately 1.92, 3.74 and 7.04 mg/L at 5, 20 and 80 mg/kg/day, respectively; importantly, these data were generated in toxicity studies rather than therapeutic cancer studies and therefore should not be interpreted as clinically relevant canine treatment exposures [25].

In the present study, we demonstrate that regorafenib exerts a clear concentration-dependent reduction in the metabolic potential of D17 canine osteosarcoma cells, consistent with its reported multikinase inhibition of tumour-supporting pathways. For example, regorafenib demonstrated strong, dose-dependent antitumour activity in eight gastric cancer PDX models, significantly inhibiting tumour growth in all models and consistently reducing angiogenesis, while also lowering proliferation and promoting apoptosis [26]. These preclinical findings reported by Huynh et al. support the concept that regorafenib, through its broad multikinase inhibitory activity, disrupts tumour-supporting signalling pathways, consistent with the concentration-dependent reduction in metabolic activity observed in our study in canine D17 osteosarcoma cells.

The regorafenib IC_50_ in D17 cells (15.7 ± 0.25 µM) was lower than the values previously determined in MG-63 (26 µM) and APR1 cells (42 µM) [27]. These findings fall within the broader range of responses reported in human osteosarcoma models, where biological heterogeneity, model-specific characteristics and experimental conditions may contribute to variability and limit direct comparisons [28,29,30].

Viewed from a broader perspective, these findings reinforce the value of comparative oncology, as integrating evidence across species can provide a more comprehensive understanding of the biological diversity underlying therapeutic responses and enhance the translational relevance of preclinical research. Such an approach is increasingly recognised as essential for improving the predictive value of preclinical models and accelerating the development of effective therapies [1,31].

The dose-dependent cytotoxicity of regorafenib in D17 cells was also evident in the apoptosis analysis. At the sublethal IC_25_ concentration, regorafenib largely preserved overall cell viability and did not markedly alter the proportions of apoptotic or necrotic cells. In contrast, treatment at the IC_50_ concentration significantly reduced viability and shifted the cell population towards late apoptosis and necrosis. These findings are consistent with the previously reported pro-apoptotic effects of regorafenib in human osteosarcoma models. However, whereas human osteosarcoma cells exhibited both early and late apoptotic responses [27,29,30], D17 cells accumulated predominantly in the late apoptotic and necrotic fractions. This suggests that, although regorafenib activates broadly similar cell death pathways, the kinetics and relative contribution of individual stages of cell death may differ between cellular models.

Importantly, although BAM15 pretreatment alone did not induce cytotoxicity, it altered cell-cycle distribution by decreasing the proportion of cells in S-phase and increasing their accumulation in G0/G1, suggesting a slowed cell-cycle progression rather than cell death. When combined with regorafenib, however, BAM15 enhanced the cytotoxic response, particularly at the IC_50_ concentration. Combined treatment with BAM15 and regorafenib at IC_50_ was associated with lower percentages of cells assigned to the G0/G1, S and G2/M regions compared with regorafenib alone. Following BAM15 treatment alone, the reduced S-phase fraction coincided with lowered BAX protein abundance.

In this context, beyond its established pro-apoptotic function, BAX has also been implicated in cell-cycle regulation, with experimental evidence indicating that both BAX and BAK facilitate S-phase entry, whereas their deficiency delays G1/S progression [32,33]. Conversely, under the more cytotoxic IC_50_ conditions, reduced BAX levels and BAX/BCL-2 ratios did not correspond to the increased Annexin V^+^/PI^+^ population. However, the reduced BAX/BCL-2 ratios have previously been associated with poor chemotherapy response and an unfavourable prognosis, including in patients with acute myeloid leukaemia [34,35], supporting an association with an apoptosis-resistant phenotype.

The observation of the reduced protein ratio may reflect an adaptive response favouring survival in a subset of treated cells, although this possibility and its relationship to the observed transcriptional changes and the lack of compensatory glycolytic activation require further investigation. Given that the Western blot and Seahorse analyses were each based on only two independent biological experiments, these findings should be considered preliminary. Moreover, Jiang et al. found that low-dose BAM15 increased mitochondrial respiration and proton leak while reducing ATP production and promoting futile energy expenditure, yet did not independently alter cell-cycle distribution [36].

Consistent with its established activity as a mitochondrial uncoupler [10], BAM15 reduced OXPHOS-derived ATP production in D17 cells without inducing a compensatory increase in ECAR. Regorafenib similarly suppressed mitochondrial ATP production, whereas BAM15 pretreatment did not further enhance this reduction. Thus, the greater cytotoxicity of the combined treatment cannot be explained by a quantitatively greater decline in OXPHOS-derived ATP at the analysed time point. Instead, BAM15 may have impaired the metabolic flexibility and adaptive capacity of D17 cells, making them less able to withstand regorafenib-induced stress. This interpretation is consistent with previous findings in AML cells, where BAM15 enhanced the anticancer effects of cytarabine, potentially through disruption of ROS homeostasis [37].

Moreover, shifts in the cell-cycle profile, together with the increased late apoptotic and necrotic fractions, are consistent with an impaired capacity of D17 cells to maintain proliferation and adapt to regorafenib-induced metabolic stress. This interpretation is supported by previous findings in breast cancer cells, in which BAM15-mediated mitochondrial uncoupling reduced ATP production and glycolytic capacity, suppressed proliferation, and induced apoptosis [38].

These findings suggest that combining regorafenib with BAM15-mediated metabolic modulation may offer an approach worth exploring to improve treatment responses, although its relevance beyond D17 cells remains to be established. From this perspective, targeting tumour bioenergetics may improve responses to anticancer agents without increasing their doses, an approach explored in oncology as metabolic sensitisation [10,37,38].

In our study, BAM15 and regorafenib, used as individual treatments, significantly impaired mitochondrial oxidative phosphorylation in D17 cells, while ATP production supported by glycolysis remained unaffected. This finding does not reflect a classical metabolic switch toward enhanced glycolysis, but rather preservation of glycolytic flux sufficient to maintain energy homeostasis during partial mitochondrial dysfunction [39]. Such maintenance of ATP production in the setting of reduced oxidative phosphorylation has been described in cancer cells as a manifestation of metabolic plasticity, allowing survival under therapeutic or environmental stress [17,40].

Analysis of apoptosis- and survival-related transcripts revealed a complex, concentration-dependent response that did not uniformly parallel the observed phenotypic effects. Regorafenib at IC_50_ induced the most pronounced increase in the BAX/BCL-2 ratio, whereas BAM15 pretreatment partially attenuated this transcriptional shift despite enhancing the accumulation of late apoptotic and necrotic cells. Similarly, changes in genes associated with PI3K/AKT/mTOR and MAPK signalling did not indicate consistent suppression of a single survival pathway but rather suggested the engagement of heterogeneous, treatment-dependent adaptive responses, which we also identified previously using a model of human osteosarcoma cells [27]. However, whether these signalling components contribute to the adaptation of D17 cells to regorafenib cannot be determined from transcript levels alone, as their protein abundance and phosphorylation status were not assessed.

Despite these limitations, the potential relevance of MCL-1, ERK2, mTOR and VEGFR to cellular adaptation warrants attention, particularly as they showed the most notable treatment-associated changes among the analysed transcripts. Moreover, BAM15 pretreatment increased mTOR, ERK2, and VEGFR transcript levels relative to regorafenib alone at both concentrations, indicating a reproducible transcriptional response to combined metabolic and pharmacological stress. Given the involvement of mTOR-, ERK-, and VEGFR-related signalling in cellular survival, growth, and stress adaptation, the observed transcript-level changes may reflect an attempted compensatory response to treatment [41,42,43]. Our previous studies in osteosarcoma models demonstrated that regorafenib suppresses ERK- and AKT/mTOR-related signalling mainly by reducing protein abundance or phosphorylation rather than by uniformly downregulating the corresponding transcripts [27]. Compensatory transcriptional upregulation of mTOR, ERK2, and VEGFR in BAM15-pretreated cells could therefore represent a feedback response aimed at restoring regorafenib-suppressed survival signalling. This interpretation is consistent with the bypass activation of pro-survival pathways previously implicated in regorafenib resistance, including reactivation of RAS/RAF/ERK signalling [44]. More broadly, cancer cells can compensate for pharmacological inhibition of one signalling pathway by activating alternative survival mechanisms, including reciprocal crosstalk between the MAPK and PI3K/AKT/mTOR pathways, thereby limiting the effectiveness of targeted therapies [45].

In turn, MCL-1 displayed a concentration-dependent pattern: BAM15 pretreatment reduced its expression relative to regorafenib alone at IC_25_ but increased it at IC_50_. As an anti-apoptotic member of the BCL-2 family, MCL-1 preserves mitochondrial integrity by restraining pro-apoptotic effectors, including BAX and BAK, and may consequently enable cancer cells to withstand therapeutic stress [46]. Its increased expression under BAM15/IC_50_ treatment conditions may also indicate an adaptive response of the surviving cell population. However, the biological and clinical significance of MCL-1 appears to be highly context-dependent. Mittal et al. reported that, despite its established anti-apoptotic function and association with chemoresistance, high MCL-1 expression in colorectal tumours was associated with more favourable clinical outcomes, including longer time on regorafenib treatment [47].

However, based on the observed transcriptional pattern in this study, we identify MCL-1, mTOR, ERK2, and VEGFR as candidate components of a compensatory survival and stress-adaptation program induced by combined BAM15 and regorafenib treatment at IC_50_. Undoubtedly, the functional involvement of these markers requires further validation at the protein level, including assessment of pathway activation through phosphorylation analyses.

In conclusion, this exploratory study suggests that BAM15 may modify the response of D17 canine osteosarcoma cells to regorafenib. Combined treatment produced a significantly greater reduction in cell metabolic activity than regorafenib alone at IC_50_, but not at IC_25_. It should be emphasised that this finding does not establish pharmacological sensitisation or synergism; nevertheless, the additional effect observed at IC_50_ warrants potential drug-interaction analysis, also across a broader range of BAM15 concentrations. In our study, BAM15 was tested at a single concentration of 20 mM, without a dose–response analysis. Moreover, BAM15 remains a preclinical compound and has not entered formal clinical trials in humans [48]; consequently, human pharmacokinetic, dose-ranging and systemic safety data are currently unavailable. To our knowledge, canine-specific in vitro data on BAM15 remain limited, while in vivo pharmacokinetic and safety data are also lacking, making it difficult to compare our findings with previous studies and assess potential translational relevance.

Another limitation of the current study is that it relies on a single cell line derived from a pulmonary metastasis and therefore cannot capture the diversity of treatment responses across osteosarcomas with different biological and metabolic characteristics. Nevertheless, because D17 cells originated from a pulmonary osteosarcoma metastasis, this model is relevant for exploring cellular responses to systemic anticancer agents in metastatic disease, which remains a major therapeutic challenge in osteosarcoma [15]. However, further studies across a broader concentration range, additional canine and human osteosarcoma models, and non-malignant cells are needed to assess the reproducibility, selectivity and wider relevance of these observations.

Mechanistic studies should examine the protein abundance and phosphorylation status of relevant signalling components, as the transcript-level changes reported here do not establish pathway activation. Particular attention should be given to metabolic adaptation and the possible roles of MCL-1, ERK and VEGFR signalling. In addition, the RT-qPCR findings should be confirmed using multiple reference genes validated under the experimental conditions used. Normalisation to GAPDH alone, without validation against additional reference genes, represents a limitation, particularly given its potential sensitivity to metabolic perturbations. Nevertheless, within these limitations, the presented findings provide a starting point for investigating how mitochondrial uncoupling may influence osteosarcoma responses to targeted therapy.

## 4. Materials and Methods

### 4.1. Cell Culture

The cell line used for the in vitro experiment was a well-established D17 canine osteosarcoma cell line derived from a lung OSA metastasis with an epithelial morphology. All experiments were performed using D17 cells from the same batch at passage 12. The cells showed similar growth kinetics, with a population doubling time of 14.2 ± 2.5 h (mean ± SD, based on three independent measurements). The cell line was obtained from the American Type Culture Collection (ATCC; CRL-1427). The culture conditions include Complete Growth Medium (CGM) consisting of Eagle’s Minimum Essential Medium (EMEM, Sigma-Aldrich/Merck, Poznań, Poland) with 10% Fetal Bovine Serum (Gibco, Thermo Fisher Scientific, Waltham, MA, USA) and 1% Penicillin-Streptomycin (Sigma-Aldrich/Merck, Poznań, Poland). Cells were incubated at 37 °C with an atmosphere containing 5% CO2 in a humidified incubator under constant sterile conditions. The growth medium was replaced every 2 to 3 days, and cells were passaged at 70% confluence using the Stable-Cell Trypsin solution (Sigma Aldrich/Merck, Poznań, Poland). The cells have been tested for Mycoplasma contamination by IDEXX BioAnalytics (Kornwestheim, Germany), and all cultures used in the experiments were confirmed to be Mycoplasma-free.

### 4.2. Preparation of Regorafenib and BAM15

Regorafenib (MedChemExpress, Monmouth Junction, NJ, USA) was dissolved in sterile DMSO to prepare a 10 mM stock solution, while BAM15 (Sigma-Aldrich/Merck, Poznań, Poland) was prepared as a 20 mM stock. Both solutions were filtered through 0.22 μm syringe filters and diluted in culture medium immediately prior to use.

### 4.3. Screening Assay

Regorafenib cytotoxicity was evaluated using the MTS assay (Abcam, Cambridge, UK) applying the manufacturer’s protocol. D17 cells were seeded in 96-well plates (1 × 10^4^ cells/well) in 200 μL of complete growth medium (CGM) and treated with regorafenib (0.1, 0.25, 0.5, 1, 2.5, 5, 10, 25, 50, 100 µM). Each regorafenib concentration was accompanied by a vehicle control containing the corresponding final DMSO concentration, ranging from 0.001% to 1% (*v*/*v*). After 48 h of drug exposure, MTS reagent was added to the cells, and absorbance was measured at 490 nm using a Spark 10M plate reader (Tecan, Männedorf, Switzerland). The IC_50_ value was calculated as 15.7 ± 0.25 µM. BAM15 was tested at a single concentration of 20 µM, selected with reference to the study by Axelrod et al. [13]. Preliminary experiments assessing this specific concentration showed no significant cytotoxic effect of BAM15 alone, as evaluated by metabolic activity and Annexin V/PI assays.

### 4.4. Experiment Design

Cells were seeded at 8 × 10^3^ cells/well on a 96-well plate and allowed to adhere for 24 h. Treatment groups included: (1) vehicle control (DMSO), (2) regorafenib IC_25_ (8 µM), (3) regorafenib IC_50_ (16 µM), (4) BAM15 alone (20 µM), (5) BAM15 + regorafenib IC_25_, and (6) BAM15 + regorafenib IC_50_. The vehicle control contained 0.26% DMSO by volume, corresponding to the highest final DMSO concentration among the treatment groups, present in the BAM15 + regorafenib IC_50_ combination. Final DMSO concentrations in the other treatment groups varied by compound and dose.

Cultures were pretreated with BAM15 for 16 h, followed by regorafenib exposure for 48 h.

### 4.5. Cell Viability Assays

Cell viability was assessed using the MTS assay according to the manufacturer’s instructions (Abcam, Cambridge, UK). Three independent biological experiments were performed, each with three technical replicates per condition. Results were normalised to vehicle-treated controls.

### 4.6. Seahorse Extracellular Flux Analysis (OCR/ECAR)

Mitochondrial respiration and glycolytic activity were assessed using a Seahorse XFe96 Analyser (Agilent Technologies, Santa Clara, CA) applying the manufacturer’s protocol and previously published methodology [27]. Prior to analysis, cells were incubated for one hour in Seahorse XF DMEM (Agilent Technologies, Santa Clara, CA, USA) (pH 7.4) supplemented with glucose (10 mM), pyruvate (1 mM), and glutamine (2 mM). The sensor cartridge was prepared according to the manufacturer’s instructions, with inhibitors loaded to achieve final assay concentrations of 1.5 µM oligomycin and 0.5 µM each of rotenone and antimycin A. The resulting Seahorse data were processed and analysed using Wave software (version 2.6; Agilent Technologies, Santa Clara, CA, USA). Oxygen consumption rate (OCR) and extracellular acidification rate (ECAR) were measured using the Mito Stress Test and Glycolysis Stress Test protocols with sequential injections of oligomycin, FCCP, and rotenone/antimycin A. Data were analysed using Wave software. Prior to exporting the dataset, ATP production rate and metabolic parameters were normalised to the cell number per well using the Normalise module in the Wave software. These normalised values were then used for subsequent analyses and graphical representations.

### 4.7. Apoptosis Profile Assays and Cell Cyle Analysis

Apoptosis was assessed by Annexin V/PI staining using the Dead Cell Apoptosis Kit (Invitrogen Life Technologies, Warsaw, Poland). Following washing, the cells were resuspended in 100 µL of 1X annexin-binding buffer. Subsequently, 5 µL of Alexa Fluor™ 488 Annexin V and 1 µL of propidium iodide (PI) working solution (100 µg/mL) were added to the suspension. The reagents are part of the Dead Cell Apoptosis Kit. The samples were then incubated at room temperature for 15 min in the dark. After incubation, 400 µL of 1x annexin-binding buffer was added to the samples, gently mixed, and then kept on ice until analysis. Samples were analysed using excitation at 488 nm and emission detection at 530 nm (Annexin V) and 575 nm (PI).

For each sample, 30,000 events corresponding to individual cells were acquired. Cellular debris was excluded according to forward and side scatter properties, and cell aggregates were eliminated by comparing FSC signal area with height and width parameters. Subsequent analysis was restricted to the singlet population, after which quadrant gating was applied to evaluate apoptosis. Subsequent analysis was restricted to the singlet population. Quadrant gating was then applied to identify viable (Annexin V^−^/PI^−^), early apoptotic (Annexin V^+^/PI^−^), late apoptotic/secondary necrotic (Annexin V^+^/PI^+^), and Annexin V^−^/PI^+^ cells, consistent with primary necrosis. Gates were defined based on unstained and single-stained reference controls and were consistently maintained across all experimental groups.

For cell-cycle analysis, we assessed DNA content using FxCycle™ PI/RNase Staining Solution (Invitrogen Life Technologies, Warsaw, Poland). Following trypsinisation, cells were fixed in 70% ethanol for 24 h at 4 °C. After washing to remove the fixative, samples were centrifuged at 500× *g* for 5 min at 4 °C. Cell pellets were resuspended in 0.5 mL of FxCycle™ PI/RNase Staining Solution, gently mixed and incubated for 30 min at room temperature in the dark. Samples were analysed using 488-nm excitation and a 585/42-nm bandpass emission filter.

For stained cell-cycle samples, 10,000 total events were recorded per acquisition. After debris exclusion and singlet gating, DNA-content histograms were used to identify the G0/G1, S, and G2/M regions, with untreated controls as a reference. Percentages were calculated relative to the parent population.

The assays were performed according to the manufacturer’s instructions and previously described procedures [27,49]. Measurements were performed using a CytoFLEX flow cytometer (Beckman Coulter, Brea, CA, USA) while data were processed using CytExpert 2.4 software.

### 4.8. Cancer-Related Gene Expression Analysis

Gene expression measurements were performed through quantitative real-time PCR using the SensiFAST SYBR^®^ & Fluorescein Kit (Bioline Reagents Ltd., London, UK) on a CFX Opus 384 Real-Time PCR Detection System (Bio-Rad, Hercules, CA, USA). Total RNA was extracted using TRI Reagent (Sigma-Aldrich/Merck, Poznań, Poland) following the manufacturer’s instructions. RNA concentration and purity were assessed spectrophotometrically using a DS-11 Fx spectrophotometer (Denovix, Wilmington, DE, USA).

To preserve RNA integrity, total RNA (500 ng) was treated with DNase I using the PrecisionDNAse kit (PrimerDesign, BLIRT S.A., Gdańsk, Poland) prior to reverse transcription. cDNA was then synthesised using the Tetro cDNA Synthesis Kit (Bioline Reagents Limited, London, UK) according to the manufacturer’s protocol, using a T100 Thermal Cycler (Bio-Rad, Hercules, CA, USA). Each reaction was performed in a 10 µL total volume, consisting of 1 µL cDNA, 5 µL 2× Master Mix, and gene-specific primers at a final concentration of 500 nM. The thermal profile began with initial denaturation at 95 °C for 2 min, followed by 40 cycles of denaturation at 95 °C for 15 s, annealing at the primer-specific temperature (typically 62 °C for 15 s), and extension at 72 °C for 15 s.

Gene expression levels were normalised to the reference gene (GAPDH), and relative transcript abundance was calculated using the 2^–ΔΔCq^ method [50], incorporating the RQ_MAX_ normalisation approach, in which data are scaled to the lowest-expressed reference sample as described previously [27,49]. The primer sequences applied in this study are presented in Table S1.

### 4.9. Protein Expression Analysis—Western Blot

Protein expression was analysed by Western blotting following a protocol previously established in our laboratory [27,49]. Total protein was isolated from D17 cells using ice-cold RIPA buffer (Sigma-Aldrich/Merck, Poznań, Poland) supplemented with a 1% protease and phosphatase inhibitor cocktail (Thermo Fisher Scientific, Warsaw, Poland). Protein concentration was determined using a bicinchoninic acid (BCA) assay (Thermo Fisher Scientific, Warsaw, Poland). Equal amounts (10 μg) of protein were separated by SDS-PAGE, transferred onto PVDF membranes, and incubated with the appropriate primary and HRP-conjugated secondary antibodies. The list of antibodies used is presented in Table S2. Protein bands were visualised using the ChemiDoc™ XRS imaging system (Bio-Rad, Hercules, CA, USA), and band intensities were quantified by densitometric analysis using Image Lab™ software (version 6.1; Bio-Rad: Hercules, CA, USA; 2020) and normalised to β-actin (ACTB).

### 4.10. Statistical Analysis

All data were analysed using GraphPad Prism version 10.6.1 (GraphPad Software, San Diego, CA, USA). All assays were performed using three independent biological replicates, each measured in technical triplicate, except for Seahorse and Western blot analyses, which were performed using two independent biological replicates, each measured in technical duplicate. Statistical analyses were based on values averaged within each biological replicate, following the methodology previously established by our group for comparable datasets [49]. Graphs and statistical outputs were generated within the software. Normality of data distribution was assessed using the Shapiro–Wilk test. Statistical analyses were performed using one-way ANOVA, followed by Tukey’s multiple comparisons post-hoc test. Differences that were not statistically significant were denoted as “ns”. A significance threshold of *p* < 0.05 was applied. Statistical significance was indicated in the figures with asterisks as follows: *p* < 0.05 (*), *p* < 0.01 (**), *p* < 0.001 (***), and *p* < 0.0001 (****).

## Data Availability

The source data supporting the conclusions of this article are openly available in the Repository of the Wrocław University of Environmental and Life Sciences at [DOI: 10.57755/bb2h-ky62] for the RT-qPCR data and at [DOI: 10.57755/8850-1564] for the original Western blot data. In addition, the data supporting this study are included in the article and its Supplementary Materials. Further inquiries can be directed to the corresponding author.

## Supplementary Materials

The following supporting information can be downloaded at https://www.mdpi.com/article/10.3390/ijms27209121/s1.
